# CXCL12 and CXCL13 Cytokine Serum Levels Are Associated with the Magnitude and the Quality of SARS-CoV-2 Humoral Responses

**DOI:** 10.3390/v14122665

**Published:** 2022-11-28

**Authors:** Alessandra Noto, Victor Joo, Antonio Mancarella, Madeleine Suffiotti, Celine Pellaton, Craig Fenwick, Matthieu Perreau, Giuseppe Pantaleo

**Affiliations:** 1 Service of Immunology and Allergy, Lausanne University Hospital, University of Lausanne, 1011 Lausanne, Switzerland; 2 Swiss Vaccine Research Institute, Lausanne University Hospital, University of Lausanne, 1011 Lausanne, Switzerland

**Keywords:** SARS-CoV-2, memory B cells, cytokines

## Abstract

A better understanding of the immunological markers associated with long-lasting immune responses to SARS-CoV-2 infection is of paramount importance. In the present study, we characterized SARS-CoV-2-specific humoral responses in hospitalized (ICU and non-ICU) and non-hospitalized individuals at six months post-onset of symptoms (POS) (N = 95). We showed that the proportion of individuals with detectable anti-SARS-CoV-2 IgG or neutralizing (NAb) responses and the titers of antibodies were significantly reduced in non-hospitalized individuals, compared to ICU- or non-ICU-hospitalized individuals at 6 months POS. Interestingly, SARS-CoV-2-specific memory B cells persist at 6 months POS in both ICU and non-ICU patients and were enriched in cells harboring an activated and/or exhausted phenotype. The frequency/phenotype of SARS-CoV-2-specific memory B cells and the magnitude of IgG or NAb responses at 6 months POS correlated with the serum immune signature detected at patient admission. In particular, the serum levels of CXCL13, IL-1RA, and G-CSF directly correlated with the frequency of Spike-specific B cells and the magnitude of Spike-specific IgG or NAb, while the serum levels of CXCL12 showed an antagonizing effect. Our results indicate that the balance between CXCL12 and CXCL13 is an early marker associated with the magnitude and the quality of the SARS-CoV-2 humoral memory.

## 1. Introduction

Severe acute respiratory syndrome coronavirus 2 (SARS-CoV-2) is currently responsible for a global pandemic, with a death toll of 6.5 million people worldwide who had been infected, as of early October 2022 [1]. The efficacy of licensed SARS-CoV-2 vaccines ranges from 50 to 95%, depending on the vaccine type and infection variant [2,3]. Despite the introduction of new COVID-19 vaccines, more than 3.5 million deaths due to COVID-19 have been reported globally since the first vaccine was administered [4]. Although the majority of infected people are asymptomatic or show mild symptoms, nearly 15–30% of the infected individuals progress to severe coronavirus disease 2019 (COVID-19) and develop acute pneumonia [5,6,7].

The pathogenicity of COVID-19 is complex and, in the absence of vaccination or previous exposure to the SARS-CoV-2 virus, the severity of the disease has been hypothesized to result from an excessive inflammatory immune response, which may cause a life-threatening multi-organ systemic clinical syndrome [8,9,10]. The activation of macrophages, epithelial cells, and, possibly, endothelial cells is responsible for the elevated serum levels of proinflammatory cytokines (interleukins (IL)-1β and IL-18, IL-6) chemokines (CCL2, CCL3, CXCL8, CXCL9, and CXCL10, CXCL11), and growth factors (G-CSF and HGF) that contribute to the pathogenic inflammation responsible for the severity of the symptoms of COVID-19 [11,12,13].

Serological analyses have shown that SARS-CoV-2 infection elicits a strong virus-specific IgA, IgM, and IgG antibody response within 1–2 weeks after the onset of symptoms [14]. SARS-CoV-2 antibodies are mainly directed against the Nucleocapisd (N) and the Spike viral proteins [15,16]. The Spike protein is composed of S1 and S2 domains [17,18]. S1 is the membrane distal and contains the receptor-binding domain (RBD) that binds to the cellular receptor angiotensin-converting enzyme 2 (ACE2) [19,20]. Antibodies that bind to RBD can prevent interactions with ACE2, whereas those that bind to other regions of S1 and S2 can inhibit conformational changes to the S protein and, thereby, block membrane fusion [21,22,23]. Studies have indicated that COVID-19 patients exhibit a strong neutralizing antibody response, the magnitude of which positively correlates with disease severity [24,25,26,27,28]. Notably, high levels of inflammatory cytokines and the presence of activated monocytes in the peripheral blood and in the lung are associated with the magnitude of the antibody responses [29,30,31,32].

Additional studies have revealed that the magnitude of SARS-CoV-2-specific B cell responses appeared to be higher in COVID-19 patients than in patients with mild or asymptomatic SARS-CoV-2 infection [33]. Interestingly, the longitudinal analysis indicated that the SARS-CoV-2-specific B cell may peak between 3 and 6 months post-infection in recovered individuals [34,35]. However, the persistence of long-lived immunological memory regarding SARS-CoV-2 remains a matter of debate, based on the conflicting results generated from various and heterogeneous cohorts [14,16,36,37].

In the context of the continual emergence of SARS-CoV-2 variants (as reviewed in [38,39]), efforts to understand how the immune system helps to control severe infection from the SARS-CoV-2 virus, leading to tissue damage, organ failure, or the death of the host, are still necessary. The comprehensive qualitative and quantitative characterization of the SARS-CoV-2 humoral responses and a better understanding of the humoral immune response in individuals who have recovered from SARS-CoV-2 infection is also required in the light of strategies that should be adopted for the vaccine-based immunity that needs to be created to avoid the spread of new variants [40]. In addition, the identification of immunological parameters shaping the emergence of a protective immune response remains to be addressed. Therefore, in the present study, we first characterized the persistence of specific antibodies against SARS-CoV-2, as well as the memory B cell responses to the Spike, RBD, and N proteins in a cohort of individuals who had recovered from severe or mild SARS-CoV-2 infections. In addition, we identified the immunological parameters that were associated with the emergence, magnitude, and quality of SARS-CoV-2 antibody and memory B cell responses.

## 2. Materials and Methods

### 2.1. Study Population

The study population includes patients with documented SARS-CoV-2 (positive PCR) infection who were admitted to the Lausanne University Hospital by the dedicated COVID-19 team of the Infectious Diseases Service or the physicians of the departments of Internal Medicine or Adult Intensive Medicine. Follow-ups of the convalescent samples were performed 6 months after recruitment for those participants whose acute illness was resolved or those who were discharged from the hospital before either of these time points came to pass. All patients gave written informed consent and were ≥18 years old.

### 2.2. CyTOF Marker Labeling and Detection

Cryopreserved peripheral blood mononuclear cells (PBMCs) were thawed and resuspended in complete RPMI medium (Gibco; Life Technologies, Carlsbad, CA, USA; 10% heat-inactivated FBS (Institut de Biotechnologies Jacques Boy, Reims, France), 100 IU/mL penicillin, and 100 μg/mL streptomycin (BioConcept, Allschwil, Switzerland)) and cells were stained as previously described [41,42]. Briefly, cells were washed in PBS and then incubated for 30 min at 4 °C, with S, RBD, and N biotinylated proteins bound to a streptavidin PE, APC, and FITC, together with a 50 μL antibody cocktail of cell surface metal-conjugated antibodies (Fluidigm/DVS Science, South San Francisco, CA, USA). Cells were washed and fixed for 10 min at room temperature (RT) with 2.4% PFA. The total cells were identified via DNA intercalation (1 μM Cell-ID Intercalator, Fluidigm/DVS Science) in 2% PFA at 4 °C overnight. The list of metal isotope antibodies used is listed in Supplementary Table S1. Labeled samples were assessed using a CyTOF1 instrument that was upgraded to CyTOF2 (Fluidigm /DVS Science, South San Francisco, CA, USA), using a flow rate of 0.045 mL/min.

### 2.3. CyTOF Data Analysis

FCS files were normalized to the EQ Four Element Calibration Beads using the CyTOF software. For conventional cytometric analysis of B cell populations, FCS files were imported into Cytobank Data Analysis Software or FlowJo v10.4.2 (Treestar, Inc., Ashland, CA, USA).

### 2.4. ELISpot Assay

PBMCs were stimulated or not for 5 days with 1 ug/mL of R848 (InvivoGen, San Diego, CA, USA) and 10 ng/mL of IL-2 (Miltenyi Biotec, Bergisch Gladbach, Germany). ELISPOT plates (BD) were coated with 15 μg/mL of anti-IgG (Mabtech, Stockholm, Sweden) and stored at 4 °C overnight. Next, the plates were washed, and cells were added for 24 h at 37 °C, followed by the addition of the biotinylated antibody against IgG or biotinylated proteins, and finally, the addition of a streptavidin-HRP (Mabtech, Stockholm, Sweden). Frequencies of the SARS-CoV-2-specific antibody-secreting cells (ASC) were calculated from triplicate or duplicate wells plated with 300.000 PBMCs per well. PBMCs from the pre-pandemic samples were used as controls.

### 2.5. Luminex Anti-SARS-CoV–2 S Protein IgG Binding Assay

Preparation of the Luminex beads coupled with the Spike protein trimer was performed as previously described in [43]. Spike protein-coupled Luminex beads were added to Bio-Plex Pro 96-well flat-bottomed plates and washed with PBS, before adding 50 μL of a 1/300 dilution of individual serum to the wells. The plates were agitated at 500 rpm for 60 min on a plate shaker. The beads were then washed, and anti-human IgG-PE (OneLambda, Thermo Fisher, Waltham, MA, USA) secondary antibody (BioConcept) was added. The plates were agitated for 45 min and washed again. Beads were resuspended in 80 μL of reading buffer and read directly on a Luminex FLEXMAP 3D plate reader (Thermo Fisher). The MFI signal of each test serum sample was divided by the mean signal of 4 replicates in a pool of 100 negative pre-pandemic control samples, yielding an MFI ratio.

### 2.6. Protocol for the Evaluation of Anti-SARS-CoV-2 Neutralizing Antibodies

Luminex MagPlex^®^ microspheres were coupled with the SARS-CoV-2 Spike trimer, according to the manufacturer’s protocol, using 100 μg of PBS dialyzed protein with 1 mL of activated beads. Following the conjugation and blocking steps, the beads were washed twice with sterile PBS and stored in Bio-Rad storage buffer at 4 °C. In performing the surrogate neutralization assay, Spike-coupled beads were diluted to 1/100 in PBS, with 50 μL added to each well of a Bio-Plex Pro 96-well flat-bottomed plates (Bio-Rad, Hercules, CA, USA). Following bead-washing with PBS on a magnetic plate washer (MAG2× program), 50 μL of individual serum samples at different dilutions in PBS were added to the plate wells. Control wells were included on each 96-well plate that included beads alone, matching the serum dilutions of a control pool of pre-COVID-19 pandemic healthy human sera (BioWest human serum AB males; VWR) and a positive control of commercial anti-Spike blocking antibody (SAD-S35, from ACRO Biosystem, Newark, DE, USA ) or recombinant-produced REGN10933 neutralizing antibody, discovered and marketed by Regeneron and tested in a concentration response. The plates were agitated on a plate shaker for 60 min, then the ACE-2 mouse Fc fusion protein (either Creative Biomart or produced by the EPFL Protein Production and Structure Core Facility) was then added to each well, at a final concentration of 1 μg/mL, and agitated for a further 60 min. The beads were then washed with the magnetic plate washer, then anti-mouse IgG-PE secondary antibody (OneLambda, Thermo Fisher) was added at a 1/100 dilution, with 50 μL per well. The plates were agitated for 45 min and washed, then the beads were resuspended in 80 μL of reading buffer, then read directly on a Luminex FLEXMAP 3D plate reader (Thermo Fisher). The MFIs for each of the beads-alone wells were averaged and used as the 100% binding signal for the ACE-2 receptor to the bead-coupled Spike trimer. The MFI from the well containing the highest concentration (> 1 μg/mL) of commercial anti-Spike blocking antibody was used as the maximum inhibition signal. The percentage blocking of the Spike trimer/ACE-2 interaction was calculated using the following formula: % Inhibition = (1 − ((MFI Test dilution − MFI Max inhibition)/(MFI Max binding − MFI Max inhibition)) × 100). The concentration-response inhibition curves were generated with GraphPad Prism 8.3.0, using the non-linear three-parameter curve-fitting analysis of the log(agonist) vs. the response.

### 2.7. Assessment of Serum Immune Signatures

Serum concentrations of the cytokines and soluble cytokine receptors (IL-1α, IL-1β, IL-6, TNF-α, IL-27, IL-12p70, IFNα2, IL-10, IL-23, IL-9, IFN-γ, IL-4, IL-5, IL-13, IL-31, IL-17A, IL-21, IL-22, IL-2, IL-7, IL-15, BAFF, and TNF-β) (cytokine receptor (IL-1RA), chemokines (CCL3, CCL4, CXCL1, CXCL8, CXCL9, CXCL10, CXCL12, and CXCL13), and growth factors (NGF-β, FGF-2, HGF, LIF, SCF, and G-CSF) were defined, determined by a multiplex bead assay, as previously described [44] for each marker, based on the results obtained in the 450 sera collected from healthy individuals (mean + 2 standard deviations).

### 2.8. Statistical Analysis

GraphPad PRISM (v.8.4.3) and R (v.3.6.3) (The R Foundation for Statistical Computing) software were used to perform statistical analyses. The statistical significance (*p*-values) was obtained using a one-way ANOVA (Kruskal–Wallis test), followed by the Mann–Whitney test on four different groups (pre-pandemic controls, SARS-CoV-2-infected individual ICU, non-ICU, and non-hospitalized groups). Serum marker level values were log-10 transformed and the differences between independent groups (ICU, non-ICU, and non-hospitalized) were tested using the Kruskal–Wallis test, while *p*-values were corrected for multiple testing, using the Bonferroni method. Correlative analyses were performed on log-10-transformed frequencies, using Spearman’s rho test.

## 3. Results

### 3.1. Study Cohort

The aim of the present study was to identify early factors that could predict the severity of SARS-CoV-2 infection. To address this objective, plasma and peripheral blood mononuclear cells (PBMCs) were collected at 6 months post-onset of symptoms (POS) from 72 patients that suffered from severe COVID-19 and required hospitalization, along with 23 individuals who did not require hospitalization, referred to as “non-hospitalized individuals”. Among the 72 hospitalized patients, 31 were admitted to an intensive care unit (ICU), referred to as “ICU patients”, and 41 were admitted to the internal medicine ward, referred to as “non-ICU patients”. Notably, all non-hospitalized individuals recruited in the present study were SARS-CoV-2 antibody-positive at the recruitment date, and all hospitalized individuals were PCR-confirmed at admission. Blood and serum samples collected at 6 months POS were used to characterize the ex vivo cellular and serum immune signatures, using mass cytometry and multiplex bead assays. Clinical data of the patients enrolled in the study are summarized in Table 1.

### 3.2. Anti-SARS-CoV2 IgG and Neutralizing Antibody Responses in Hospitalized and Non-Hospitalized Individuals

**Figure 1:**
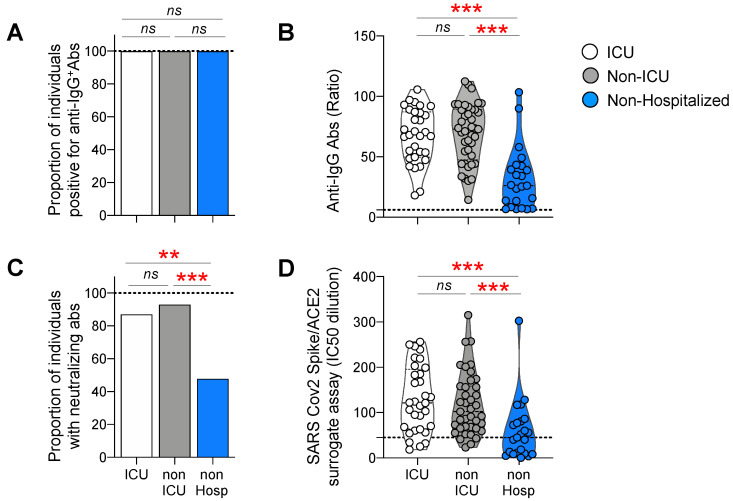
Antibody responses against the Spike protein in ICU, non-ICU, and non-hospitalized individuals. Luminex beads, coupled with Spike proteins, were used to monitor the IgG binding antibody response; neutralization was measured by a surrogate Spike/ACE-2 inhibition binding assay in the sera, from ICU (N = 31), non-ICU (N = 41), and non-hospitalized (N = 23) individuals at 6 months post-SARS-CoV-2 infection. The proportion of individuals with detectable (**A**) IgG (**C**) neutralizing antibodies in the three study groups. (**B**) Levels of IgG-binding antibody responses: MFI signals for serum antibody-binding were expressed as ratios, compared to a negative-control pool of pre-COVID-19 pandemic human serum, tested in parallel. (**D**) IC50 dilutions of the Spike/ACE2 surrogate neutralization assay. *p*-values were obtained using chi-square and a one-way ANOVA (Kruskal–Wallis test), followed by a Mann–Whitney test. Red stars indicate statistical significance (** = *p* < 0.001; *** = *p* < 0.001); *ns* = not significant.

First, we assessed the presence of anti-SARS-CoV2 IgG and neutralizing antibody (NAb) responses in the sera of hospitalized individuals (ICU and non-ICU patients) and non-hospitalized individuals, collected at 6 months POS. Notably, the levels of anti-SARS-CoV-2 IgG were assessed using a trimeric Spike-based multiplex semi-quantitative bead assay [43], while SARS-CoV-2-specific NAbs were assessed using a receptor-binding competition-based assay, as previously described [45]. The cumulative data indicated that all individuals from the three groups had detectable anti-SARS-CoV-2 IgG antibodies (Figure 1A), while the levels of anti-SARS-CoV-2 IgG antibodies were significantly reduced in non-hospitalized compared to hospitalized individuals at 6 months POS (mean IgG levels: non-hospitalized = 31.3; ICU = 67.5; non-ICU = 69.4; *p* < 0.0001) (Figure 1B). However, both the proportion and the titers of non-hospitalized individuals with anti-SARS-CoV-2 NAbs dropped at 6 months POS (47.8%, mean NAb titers = 58.7), while 87% of the ICU and 93% of the non-ICU-hospitalized individuals were still NAb-positive (mean NAb titers: ICU = 130; non-ICU = 118) (Figure 1C,D). Notably, no significant differences were observed between ICU- and non-ICU-hospitalized individuals, neither in terms of the proportion of positive results nor in terms of NAb titers (*p* > 0.05) (Figure 1C,D).

Taken together, these data indicate that non-hospitalized individuals harbor reduced anti-SARS-CoV-2 IgG and NAb responses at 6 months POS, compared to hospitalized individuals. However, no significant differences were found in the proportion and levels of anti-SARS-CoV-2 IgG and NAb responses between ICU and non-ICU-hospitalized individuals.

### 3.3. Frequencies of Spike, RBD, and N-Specific B Cell Responses

**Figure 2:**
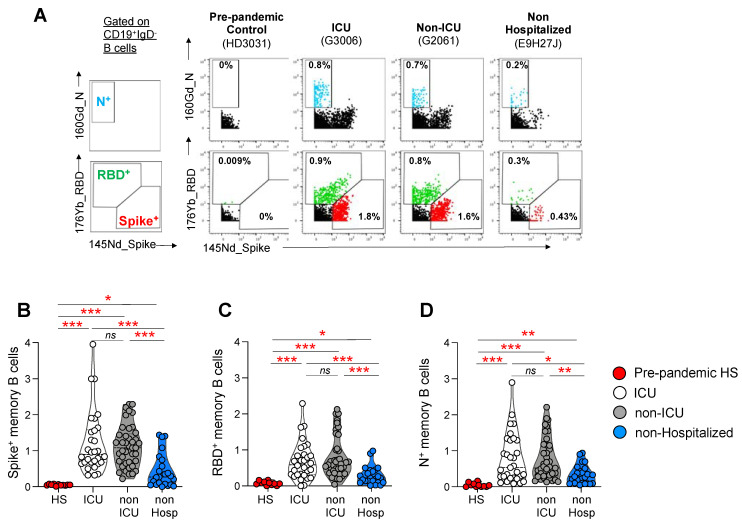
Frequencies of Spike, RBD, and N-specific B cells in ICU, non-ICU, and non-hospitalized individuals. PBMCs from ICU (N = 31), non-ICU (N = 41), and non-hospitalized (N = 23) individuals at 6 months POS and from pre-pandemic controls (N = 11) were stained with biotinylated Spike, RBD, and N proteins, which have been linked with streptavidin-conjugated metals, and a panel of 38 B cell-related markers. (**A**) Representative mass cytometry profiles of the total CD19+IgD^−^ B cell populations binding to the Spike, RBD, and N probes in one representative pre-pandemic control (HD3031), one ICU patient (G3006), one non-ICU patient (G2061) and one non-hospitalized (E9H27J) individual. (**B**–**D**) Cumulative data are shown on the frequencies of IgD-CD19+-specific B cells in the three study groups. Statistical significance (*p*-values) was obtained using a one-way ANOVA (Kruskal–Wallis test), followed by the Mann–Whitney test. Red stars indicate statistical significance (* = *p* < 0.05; ** = *p* < 0.01; *** = *p* < 0.001); *ns* = not significant.

The frequency and the immune profile of SARS-CoV-2-specific B cells were then assessed in 71 hospitalized patients (31 ICU and 41 non-ICU), 23 non-hospitalized individuals, and 11 pre-pandemic healthy subjects, using a mass cytometry panel composed of 38 markers (Supplementary Table S1). Briefly, blood SARS-CoV-2-specific B cells were identified using the biotinylated Spike, RBD, or N proteins (Figure 2A). The gating strategy is shown in Supplementary Figure S1. As is consistent with previous studies [1], Spike^+^RBD^−^ B cells are referred to as Spike^+^ B cells, and Spike^+^RBD^+^ B cells are referred to as RBD^+^ B cells, throughout the manuscript (Figure 2A). Notably, minimal binding (<0.09%) was observed in the pre-pandemic healthy controls (*p* < 0.05) (Figure 2A–D), demonstrating the specificity of the assay to thereby detect Spike, RBD, or N-specific B cells.

The cumulative data indicated that the frequencies of Spike (ICU = 1.2%; non-ICU = 1.13%, non-hospitalized = 0.44%), RBD (ICU = 0.69%; non-ICU = 0.8%; non-hospitalized = 0.28%), and N-specific (ICU = 0.78%; non-ICU = 0.74%; non-hospitalized = 0.33%) B cells were significantly reduced at 6 months POS in non-hospitalized individuals, compared to ICU or non-ICU-hospitalized individuals (Figure 2B–D). Notably, no significant differences were observed between the ICU- and non-ICU-hospitalized individuals (*p* > 0.05) (Figure 2B–D).

Overall, these data indicate that non-hospitalized individuals harbor reduced anti-SARS-CoV-2 memory B cells at 6 months POS, compared to ICU and non-ICU-hospitalized individuals. Moreover, no differences were found in the frequencies of anti-SARS-CoV-2 memory B cells at 6 months POS between ICU- and non-ICU-hospitalized individuals.

### 3.4. Immune Profile of Spike, RBD, and N^+^ Specific B Cells

**Figure 3:**
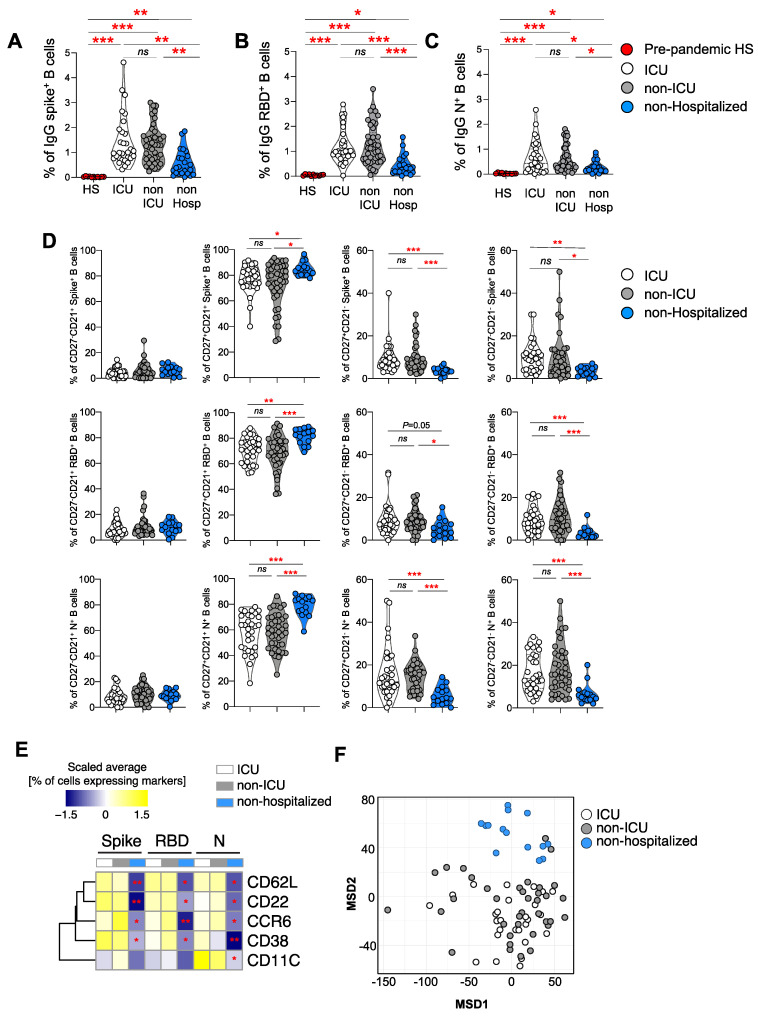
Distribution of SARS-CoV-2-specific B cells in ICU, non-ICU, and non-hospitalized individuals. PBMCs from individuals with detectable SARS-COV-2+ B cells from ICU (N = 31), non-ICU (N = 41), and non-hospitalized (N = 15) individuals at 6 months POS were profiled via mass cytometry, based on the IgG immunoglobulin isotype expression and/or by the combination of CD27 and CD21 markers identifying intermediate memory (IM; CD27^−^CD21+), classical resting memory (RM; CD27+ CD21+), activated memory (AM; CD27+CD21^−^), and tissue-like memory (TLM; CD27- CD21^−^). Frequencies of IgG+ (**A**) Spike, (**B**) RBD, and (**C**) N-specific B cells from ICU (N = 31), non-ICU (N = 41), and non-hospitalized (N = 23) individuals at 6 months POS and from pre-pandemic controls (N = 11). (**D**) Frequencies of Spike+, RBD+ and N+ B cells within the CD27^−^CD21+(IM), CD27+CD21+(RM), CD27+CD21^−^(AM), and CD27^−^CD21-(TLM) B cells. (**E**) Heat map showing scaled mean marker expression in Spike+, RBD+, and N+ B cells from ICU, non-ICU, and non-hospitalized individuals. Red stars indicate statistical significance in non-hospitalized individuals, compared to ICU and non-ICU individuals (* = *p* < 0.5, ** *p* < 0.01). (**F**) Multi-dimensional scaling (MDS) of ICU, non-ICU, and non-hospitalized individuals, based on the Spike+ B cells marker expression. *p*-values were obtained via a one-way ANOVA (Kruskal–Wallis test), followed by a Mann–Whitney test. Red stars indicate statistical significance (* = *p* < 0.5, ** *p* < 0.01, *** = *p* < 0.001); *ns* = not significant.

Blood antigen-experienced (CD19^+^IgD^−^) B cells are classically profiled by the combination of CD27 and CD21 markers identifying intermediate memory (IM; CD27^−^CD21^+^), classical resting memory (RM; CD27^+^ CD21^+^), activated memory (AM; CD27^+^CD21^−^), and tissue-like memory (TLM; CD27^−^ CD21^−^) [46]. Therefore, we first measured the frequencies of IgG+ memory B cells in the three groups. The frequencies of Spike, RBD, and N IgG^+^ B cells were significantly decreased in non-hospitalized individuals, compared to ICU- or non-ICU-hospitalized individuals (IgG^+^Spike^+^ B cells =ICU = 1.4%, non-ICU = 1.3%, non-hospitalized = 0.56%; *p* < 0.01; IgG^+^RBD^+^ B cells = ICU = 1.1%, non-ICU = 1.2%, non-hospitalized = 0.4%; *p* < 0.001; IgG^+^N^+^ B cells = ICU = 0.6%, non-ICU = 0.6%, and non-hospitalized cases = 0.2%; *p* < 0.05), while no significant differences were found between ICU- and non-ICU-hospitalized subjects (*p* > 0.05) (Figure 3A–C).

Next, we evaluated the distribution of Spike, RBD, and N-specific memory B cells, based on CD21 and CD27 expression in the three study groups in individuals with detectable memory B cells. We found that at 6 months POS, the majority of the Spike, RBD, and N-specific memory B cells were enriched within the RM B cells in all groups (Figure 3D). Of note, Spike, RBD, and N-specific memory B cells were statistically enriched within the RM B cells in non-hospitalized individuals (*p* < 0.05) (Figure 3D). However, ICU- and non-ICU-hospitalized individuals still harbored increased proportions of AM and TLM Spike, RBD, and N-specific B cells, compared to non-hospitalized individuals (*p* < 0.05) (Figure 3D). However, no significant differences were observed between ICU- and non-ICU-hospitalized individuals (*p* > 0.05) (Figure 3D). In addition, Spike, RBD, and N-specific B cells from the ICU- and non-ICU-hospitalized individuals harbored increased levels of CD62L, CD22, CD38, and CD11c B cell activation markers, compared to non-hospitalized individuals (Figure 3E). Finally, multi-dimensional scaling (MDS), used to examine the general distribution of SARS-CoV-2 memory B cells of the three study groups, showed that non-hospitalized individuals were clearly segregated away from ICU and non-ICU individuals in the MDS space, whereas ICU and non-ICU individuals largely overlapped (Figure 3F).

Together, these data suggest that the memory B cells of hospitalized individuals at 6 months POS still maintain an activated status, compared to non-hospitalized individuals. However, no differences were found in the distribution and in the profile of anti-SARS-CoV-2 memory B cells at 6 months POS between ICU- and non-ICU-hospitalized individuals.

### 3.5. Frequencies of Antibody Secreting Cells after Polyclonal Stimulation

**Figure 4:**
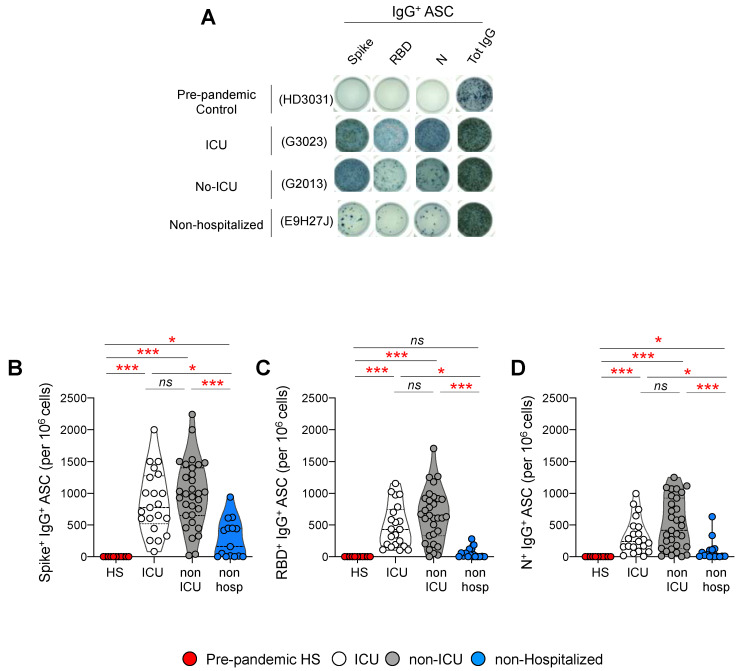
Frequencies of SARS-CoV-2+ antibody-secreting cells (ASCs) in ICU, non-ICU, and non-hospitalized individuals. PBMCs from ICU (N = 21), non-ICU (N = 31), and non-hospitalized (N = 15) individuals at 6 months POS and from pre-pandemic controls (N = 10) were cultured for 5 days in the presence of R848 (1 ug/mL) and IL-2 (10 ng/mL). The frequencies of SARS-CoV-2-specific cells (ASC) were measured by ELISPOT. (**A**) Representative counting of spot-forming cells (SFC) in representative ICU, non-ICU, non-hospitalized individuals, and one pre-pandemic control. Cumulative data on the frequencies of IgG+, (**B**) Spike, (**C**) RBD, and (**D**) N-specific ASCs in the different groups. *p*-values were obtained using a one-way ANOVA (Kruskal–Wallis test), followed by a Mann–Whitney test (intra-group comparisons). Red stars indicate statistical significance (* *p* < 0.05, *** = *p* < 0.001); *ns* = not significant.

We next investigated whether the distinct SARS-CoV-2 specific memory B cell profiles identified in hospitalized and non-hospitalized individuals may translate into an altered plasmocyte differentiation potential. To address this issue, the blood mononuclear cells of 52 hospitalized patients (21 ICU and 31 non-ICU patients), 15 non-hospitalized individuals, and 11 pre-pandemic healthy subjects were stimulated with R848 and IL-2, and the frequencies of SARS-CoV-2-specific antibody-secreting cells (ASCs) were assessed by ELISpot, as previously described [47]. Notably, no SARS-Co2 ASCs were detected in pre-pandemic healthy controls (*p* < 0.05) (Figure 4A–D), demonstrating the specificity of the assay.

The representative example and the cumulative data indicated that the frequencies of IgG^+^ Spike (ICU= 861 sfu, non-ICU= 983.6 sfu, non-hospitalized = 280 sfu), RBD (ICU= 481 sfu, non-ICU= 645 sfu, non-hospitalized = 54 sfu) and N-specific (ICU= 322 sfu, non-ICU= 510 sfu, non-hospitalized = 91 sfu;) ASCs were significantly reduced at 6 months POS in non-hospitalized individuals, compared to ICU- or non-ICU-hospitalized individuals (Figure 4B–D). Notably, no significant differences were observed between ICU- and non-ICU-hospitalized individuals (*p* > 0.05) (Figure 4B–D).

Overall, these data confirmed that non-hospitalized individuals harbored reduced anti-SARS-CoV-2 memory B cell frequencies at 6 months POS, compared to hospitalized individuals, while no differences were found between ICU- and non-ICU-hospitalized individuals.

### 3.6. Cytokine Signatures in Hospitalized and Non-Hospitalized Individuals

**Figure 5:**
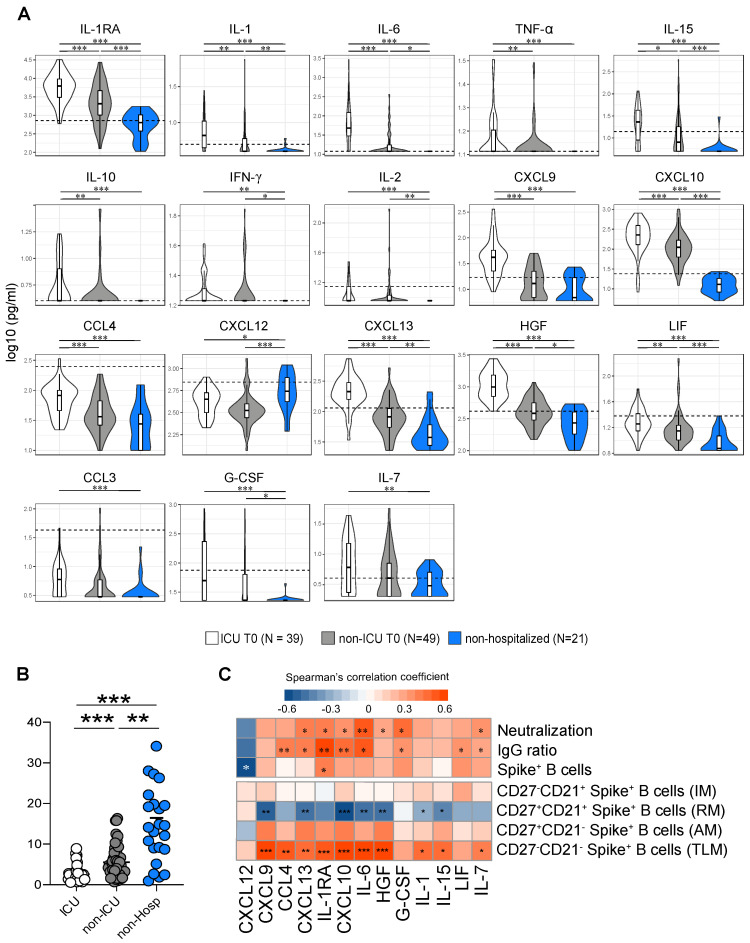
Serum cytokine, soluble cytokine receptor, chemokine, and growth factor profiles in ICU, non-ICU and non-hospitalized individuals at admission. (**A**) Levels of cytokines (IL-1α, IL-1β, IL-6, TNF-α, IL-10, IFN-γ, IL-2, IL-7, IL-15, chemokines (CCL4, CXCL9, CXCL10, CXCL12, CXCL13) and growth factors (HGH, LIF, G-CSF) in ICU (N = 39), non-ICU (N = 49) and non-hospitalized (N = 22) patients at the time of the admission. Dotted line represents the mean cytokine value calculated on serum from 450 healthy pre-pandemic controls. Stars indicate statistical significance between admission the three study groups. Statistical significance (*p*-values) was obtained using Kruskal-Wallis test, using a Bonferroni correction. * = *p* < 0.05; ** = *p* < 0.01; *** = *p* < 0.001. (**B**) CXCL12/CXCL13 ratio and (**C**) Correlative analysis between the serum cytokine levels at the time of SARS-CoV-2+ test with the frequencies of total Spike+ B cells, with the frequencies of Spike+ IM, RM, AM and TLM B cells (ICU+ non-ICU: N = 24; non-Hospitalized: N = 15) and with the IgG ratio and the neutralizing abs titers, and at 6 months POS (ICU + non-ICU: N = 24; non-Hospitalized: N = 22).

The identification of the immunological parameters associated with the emergence of protective and long-lasting immune responses is of paramount importance and they remain to be identified in SARS-CoV-2-infected individuals. We, therefore, first assessed the serum levels of a panel of cytokines, chemokines, and growth factors at admission for hospitalized individuals and at the time of serological diagnosis for non-hospitalized individuals, as well as at 6 months POS (Figures 5, S2 and S3). The cumulative data indicated that the serum levels of IL-1RA, IL-1β, IL-6, TNF-α, IL-15, IL-10, IFN-γ, IL-2, CXCL9, CXCL10, CCL4, CXCL13, HGF, LIF, CCL3, G-CSF, and IL-7 were significantly increased in ICU-hospitalized individuals, compared to non-hospitalized subjects, and IL-1RA, IL-1β, IL-6, IL-15, IFN- γ, IL-2, CXCL10, CXCL13, HGF, LIF, and G-CSF were also increased in hospitalized non-ICU individuals, compared to non-hospitalized individuals (Figure 5A). Of note, IL-1RA, IL-1β, IL-6, TNF-α, IL-15, IL-10, CXCL9, CXCL10, CCL-4, CXCL13, HGF, and LIF were increased in ICU compared to non-ICU individuals (Figure 5A). Notably, the serum levels of CXCL12 were significantly reduced in ICU- and non-ICU-hospitalized individuals, compared to non-hospitalized individuals (*p* < 0.05 and *p* < 0.001, respectively) (Figure 5A). Age and gender were assessed, but no significant differences were found among the groups.

CXCL12 and CXCL13 play an important role in antigen-specific B cell maturation within the germinal center area. Therefore, we calculated the CXCL12 versus CXCL13 ratio and found a significant decrease in ICU- and non-ICU-hospitalized individuals, compared to non-hospitalized individuals, and in the ICU compared to non-ICU group (ICU = 2.5; non-ICU = 5.5; non-hospitalized = 14.5; ** = *p* < 0.01) (Figure 5B). However, no significant differences in the cytokine levels were found in the serum at 6 months POS between the different study groups (Supplementary Figure S3).

We next performed multiparametric correlative analyses between the serum cytokine/chemokine/growth factor levels detected at admission, along with the frequency of Spike-specific B cells and the magnitude of the Spike-specific IgG or NAb responses, as well as with the SARS-CoV-2-specific B cell profiling at 6 months POS (Figure 5C). We found that the serum levels of CXCL13, IL1RA, CXCL10, IL-6, G-CSF, and IL-7 directly correlated with the magnitude of Spike-specific IgG and NAb responses at 6 months POS. Interestingly, only the IL-1RA serum levels directly correlated with the frequency of Spike-specific B cells (*p* < 0.05) (Figure 5C), while the serum levels of CXCL12 were inversely correlated with the frequency of Spike-specific B cells at 6 months POS (*p* < 0.05) (Figure 5C).

Finally, we found that the serum levels of IL-1β, IL-1RA, IL-6, IL-7, IL-15, CCL4, CXCL9, CXCL10, CXCL13, and HGF directly correlated with the proportion of TLM Spike-specific B cells at 6 months POS (*p* < 0.05) (Figure 5C), while the serum levels of IL-1β, IL-6, IL-15, CXCL9, CXCL10, CXCL13, and HGF were inversely correlated with the proportion of RM Spike-specific B cells at 6 months POS (*p* < 0.05) (Figure 5C).

Taken together, these data indicate that the serum levels of pro-inflammatory cytokines (IL-1RA, IL-1 β, IL-6, TNF-α, IFN-γ, IL-2, and IL-15), homeostatic cytokines (IL-7 and CXCL-12), chemokines (CXCL13, CCL4, CXCL9, CXCL10, and CXCL13), and growth factors (HGF, LIF, and G-CSF) specifically shape the quantitative and qualitative humoral immune responses to SARS-CoV-2 infection.

## 4. Discussion

The fine characterization of the immune signatures associated with non-hospitalized and hospitalized SARS-CoV-2-infected individuals, both at the time of admission/diagnosis and longitudinally, may help to improve the care of infected patients. In particular, a better understanding of the immunological markers associated with long-lasting immune responses to SARS-CoV-2 may help to adapt the global strategy to minimize the direct impact of COVID-19 on infected individuals and its indirect effects on the entire society.

In this context, we performed a comprehensive quantitative and qualitative characterization of the humoral responses against SARS-CoV-2 on individuals who recovered from severe COVID-19 ((individuals admitted to the intensive care unit (ICU) or admitted to the internal medicine ward (non-ICU patients)), or in non-hospitalized individuals, for up to six months post-infection.

In the present study, we first showed that the proportion of individuals with detectable anti-SARS-CoV-2 IgG or NAb responses and the titers of antibodies were significantly reduced in non-hospitalized individuals, compared to ICU- or non-ICU-hospitalized individuals at 6 months post-SARS-COV-2 infection, demonstrating the heterogeneity of the humoral immune response, depending on the severity of the infection.

This wide range of antibody titers against SARS-CoV-2 has been observed in previous studies, with higher titers in individuals with more severe disease than those with milder disease [25,27,28]. In particular, previous studies have shown that SARS-CoV-2-infected individuals develop IgM, IgA, and IgG against Spike and N within 1–2 weeks POS, which remain elevated following viral clearance [16,37,48,49,50,51,52]. Longitudinal studies have addressed the longevity of the antibody responses up to 6 and 8 months post-infection, but the information presented is still limited and controversial [34,35,53]. Some findings indicated a rapid wane of anti-SARS-CoV-2 antibody titers by 3–6 months post-infection, while other recent studies showed a stable titer over several months [25,34,35]. The differences in the results obtained might be associated with the studied populations since many studies described heterogeneous cohorts, limiting a comprehensive understanding of the immunological memory regarding SARS-CoV-2.

We next showed that anti-SARS-CoV2 IgG and NAb responses were maintained at high levels in individuals who recovered from severe COVID-19. However, no significant difference was observed between ICU- and non-ICU-hospitalized individuals at 6 months POS. These data clarify the heterogeneity of previous observations that showed either the persistence [54,55,56,57,58] or the decay [25,29] of SARS-CoV-2 antibody responses with time. These differences in the results obtained might be associated with the studied population, i.e., severe COVID-19 versus mild SARS-CoV-2 infection, healthy subjects versus immunocompromised patients [59], and based on the specificity of the assays, i.e., the use of trimeric proteins versus monomeric proteins, to detect anti-SARS-CoV-2 antibodies.

We next assessed the frequency and the profile of SARS-CoV-2 memory B cells at 6 months POS in non-hospitalized and in severe SARS-CoV-2 infection cases. We found increased frequencies of Spike^+^, RBD^+^, and N^+^ B cells in hospitalized patients, compared to non-hospitalized individuals, at 6 months POS, demonstrating the persistence of the SARS-CoV-2 immunological memory in individuals that recovered from severe COVID-19 infection.

In the context of resolved viral infections, naïve B cells respond to a T-cell-dependent non-persisting antigen through the generation of a germinal center reaction, which leads to immunoglobulin class-switching and increased affinity maturation [60]. This process culminates in the generation of long-lived resting memory B cells and plasma cells. Circulating resting antigen-specific memory B cells are characterized by the expression of specific surface markers, such as CD27 and CD21, as well as the reduced expression of markers associated with inductive or effector stages, including HLA-DR, CD38, CD95, and CD80/86. In contrast, during persistent chronic infections, several subsets, including antigen-experienced B cells, tissue-like (TLM; CD27^-^CD21^-^), and activated (AM; CD27^+^CD21^−^) virus-specific memory B cells can be identified in the peripheral blood [61]. These populations are associated with virus-induced cellular exhaustion and apoptosis [62].

Interestingly, the SARS-CoV-2-specific memory B cells of hospitalized patients were enriched in cells harboring an activated and/or exhausted phenotype, while antigen-experienced B cells from individuals who recovered from mild SARS-CoV-2 infection maintained low levels of B cell immune activation at 6 months POS.

Therefore, we investigated whether the serum levels of cytokines/chemokines and/or growth factors produced during the acute phase of the infection might be associated with the magnitude and/or the quality of SARS-CoV-2 antibody and memory B cell responses. Consistent with previous studies, a large number of serum factors were increased in hospitalized individuals, compared to non-hospitalized individuals [11,12,13] or to pre-pandemic healthy controls [63]. In our study, 70.4% of the ICU and 50.4% of the non-ICU individuals were also used in the study published by Perreau et al. [63]; similarly, we found CXCL13 to be one of the major cytokines increased during severe COVID-19 infection. However, in the work of Perreau et al., non-hospitalized individuals were not included in the study and no correlations with the humoral B cells responses were identified.

In the present study, we found that the serum levels of IL-1β, IL-1RA, IL-6, IL-7, IL-15, CCL4, CXCL9, CXCL10, CXCL13, and HGF directly correlated with the proportion of TLM Spike-specific B cells at 6 months POS. In contrast, the serum levels of IL-1β, IL-6, IL-15, CXCL9, CXCL10, CXCL13, and HGF were inversely correlated with the proportion of RM Spike-specific B cells at 6 months POS. These data indicated that the cytokine/chemokine/growth factor environment that was produced during the acute phase of SARS-CoV-2 infection is associated with the phenotypic abnormalities observed in individuals who recovered from severe COVID-19. As previously shown, the TLM subset in severe COVID-19 increases over time, while the RM subset proportionally decreases [64,65,66]; since TLM are a subset of the effector memory B cells, our data, which show a positive correlation with the proinflammatory cytokine profile, are in accordance with previous studies.

Interestingly, the serum levels of CXCL13, CXCL10, IL-1RA, and G-CSF directly correlated with the magnitude of Spike-specific IgG or NAb responses at 6 months POS. In contrast, the serum levels of CXCL12 were inversely correlated with the frequency of Spike-specific B cells at 6 months POS.

The release of G-CSF has been previously shown to correlate with the increased survival of germinal center (GC) B cells and to enhance the secretion of IgG and IgM [67,68]. A more recent clinical study has shown that in the stem cell mobilization of PBMCs, treatment with G-CSF also greatly increases the proportion of mature and memory B cells [69]. The increasing levels of G-CSF from non-hospitalized to non-ICU and ICU individuals may reflect the enhancement of humoral B cell response during severe COVID-19.

IL-1RA acts as an antagonist to IL-1R1 and IL-1R2 and suppresses early immune activation and inflammation by competing with IL-1. As previously shown, we found increased IL-1RA at the early stages of severe COVID-19 infections [70]. The increased IL-1RA levels may represent a compensatory response to the highly inflammatory status of severe cases, which, in parallel, is associated with increased Spike-specific memory B cells.

CXCL12 and CXCL13 play an important role in B-cell positioning within the germinal center area. In particular, CXCL13 plays a central physiological role in the organization of the secondary lymphoid tissue structure of primary and secondary follicles and, thus, of B cell maturation [71]. CXCL13 is a pro-inflammatory cytokine involved in several pathological conditions; the finding of increased levels in tissue and/or in serum corresponds to varying degrees of inflammation. Interestingly, increased serum levels and tissue expression of CXCL13 have initially been found to be associated with idiopathic pulmonary fibrosis [72,73] and also, recently, in several interstitial lung diseases, including idiopathic interstitial pneumonia and interstitial pneumonia with autoimmune features [74]. The increased levels of CXCL13 are associated with severe prognosis and increased mortality in all interstitial lung diseases [75,76].

Whereas it is well documented that CXCL13 levels are upregulated during severe COVID-19 infections [71,72,73,74,75,76], less is known regarding the role of CXCL12 during COVID-19, and conflicting data are available [77,78,79]. CXCL12 has been traditionally identified as a homeostatic chemokine that plays an essential role during development and is critical for the homeostatic regulation of leukocyte trafficking [80]. In addition, CXCL12 is involved in the entry of long-lived plasma cells in the appropriate bone marrow (BM) niches and plays important roles in the germinal center reaction during the immune response. In the GCs, two histologically distinct areas are observed (the dark zone (DZ) and the light zone (LZ)); the balance between CXCL12 (which is more expressed in the DZ) and CXCL13 (which is more expressed in the LZ) regulate the response of the GC B cells [81,82]. With regard to CXCL12 and CXCL13, the early modulation of these two chemokines in the ICU and non-ICU individuals may reflect the potent host immune response to promote the maturation of B cells and antibody response, in order to achieve the rapid control of virus replication and virus clearance. However, excessive CXCL13 levels are probably associated with an inadequate immune response that might be associated with severe COVID-19, while the reduced CXCL12 levels in hospitalized patients might be associated with an increased consumption by GC B cells, indicating a massive B cell response.

Collectively, our results suggest that the balance between CXCL12 and CXCL13 may be an early predictive marker associated with the magnitude and the quality of SARS-CoV-2 antibody and memory B cell responses.

## Figures and Tables

**Table 1:** Participant characteristics.

	ICU (N = 61)	Non-ICU (N = 109)	Non-Hospitalized (N = 23)
Age (years) (Median )	60 (23 to 82) IQR * = 13	60(27 to 96) IQR = 24	49.8(14.9 to 88.5) IQR = 39.1
Gender			
Female (%)	27.8% (17/61)	41.2% (45/109)	52.1% (12/23)
Male (%)	72.1% (44/61)	58.7% (64/109)	47.8% (11/23)
Sars-CoV-2 related symptoms (%)	100% (61/61)	100% (109/109)	74% (17/23)
Comorbidities (%)	72.1% (44/61)	81.6% (89/109)	n/a **
Hospitalization Status			
Never (%)	0% (0/61)	0% (0/109)	100% (23/23)
Intensive Care Unit (ICU)(%)	100% (61/61)	0% (0/109)	0% (0/23)
Internal medicine ward (%)	0% (0/61)	100% (109/109)	0% (0/23)

* IQR = Interquartile range, ** n/a = not applicable.

## Data Availability

The relevant data are within the paper and in the Supplementary Materials.

## Supplementary Materials

The following supporting information can be downloaded at: https://www.mdpi.com/article/10.3390/v14122665/s1, Table S1: Mass Cytometry panel, Figure S1: Gating strategy used to identify Spike, RBD or Nucleocapside (N)-specific memory B cells. Figure S2: Serum cytokine, soluble cytokine receptor, chemokine and growth factor profiles in ICU, non-ICU and non-hospitalized individuals at admission. Figure S3: Levels of cytokines, cytokine receptor, chemokines and growth factors at 6 months post infection in ICU, non-ICU and non-hospitalized at admission.
