# Supplementary material for: CXCL12 and CXCL13 Cytokine Serum Levels Are Associated with the Magnitude and the Quality of SARS-CoV-2 Humoral Responses
Source: Viruses. 2022 Nov 28;14(12):2665. doi: 10.3390/v14122665 (PMC9785906; doi:10.3390/v14122665)
Supplement: Supplementary file 1 [file viruses-14-02665-s001.zip › viruses-1997376-supplementary.pdf]

Supplementary figure S1

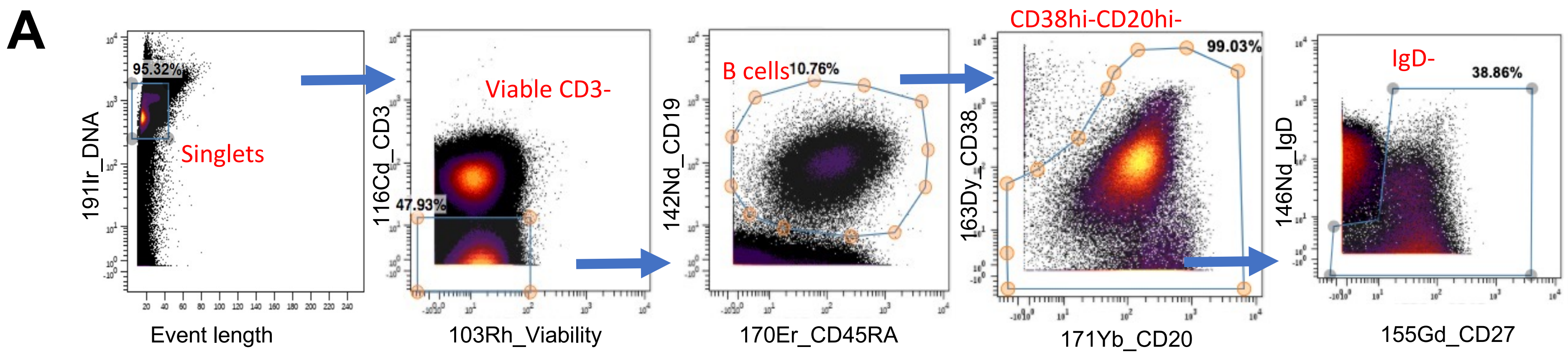

Supplementary Figure S1: Gating strategy used to identify Spike, RBD or Nucleocapside (N)-specific memory B cells.

Supplementary figure S2

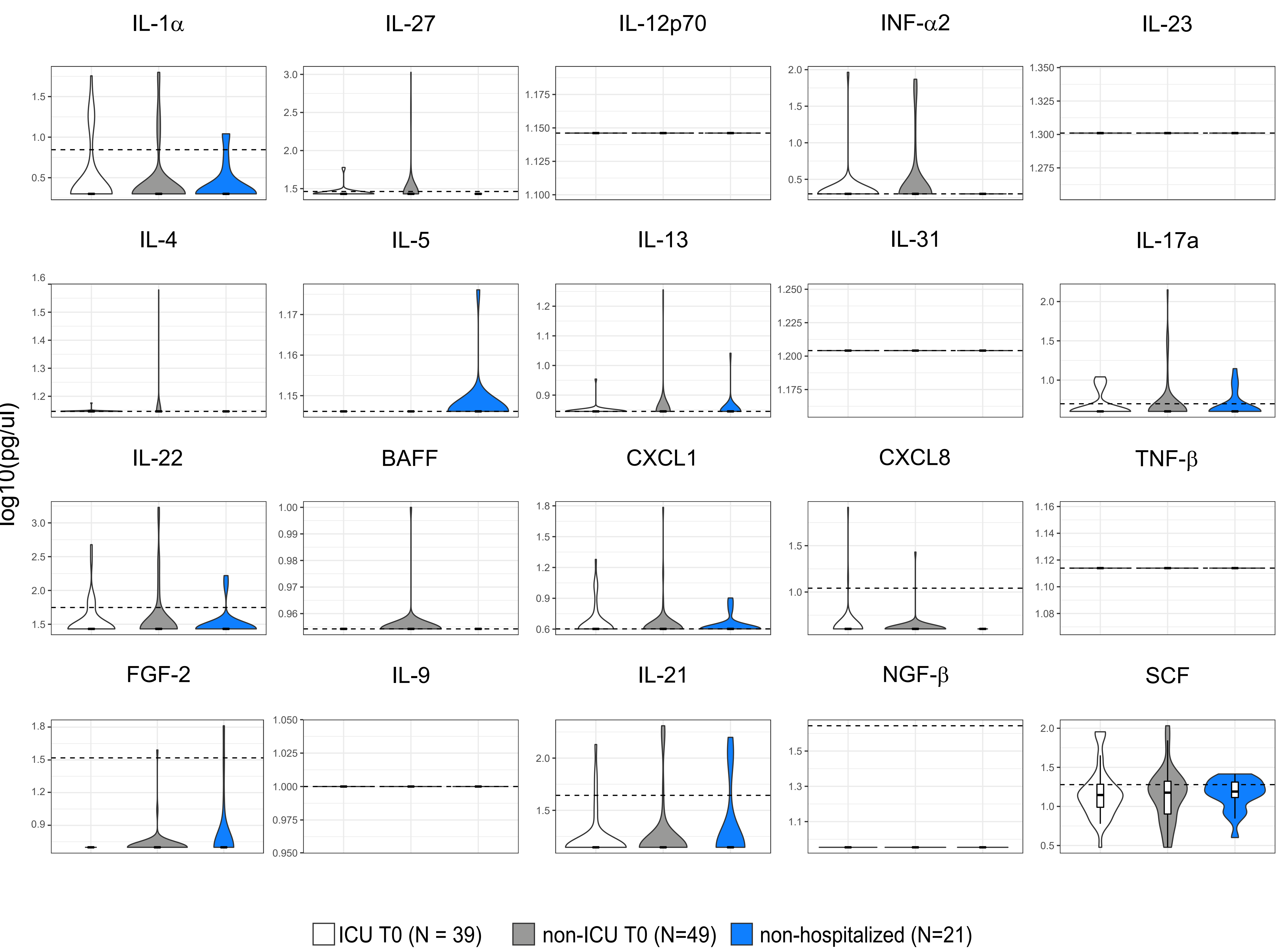

Supplementary Figure S2: Serum cytokine, soluble cytokine receptor, chemokine and growth factor profiles in ICU, non-ICU and non-hospitalized individuals at admission. (A) Levels of IL-1α, IL-27, IL-12p70, IFNα, IL-23, IL-4, IL-5, IL-13, IL-31, IL-17A, BAFF, CXCL1, CXCL8, TNF-α , FGF-2, IL-9, IL-21, NGF-b, SCF, IL-7, CCL3) in ICU (N=39), non-ICU (N=49) and non-hospitalized (N = 22) patients at the time of the admission. Dotted line represents limit of detection. Stars indicate statistical significance between the three study groups at the time of SARS-CoV-2+ diagnosis. Statistical significance (*P* values) was obtained using Kruskal-Wallis test, using a Bonferroni correction. *=*P*<0.05; **=*P*<0.01; ***=*P*<0.001.

Supplementary figure S3

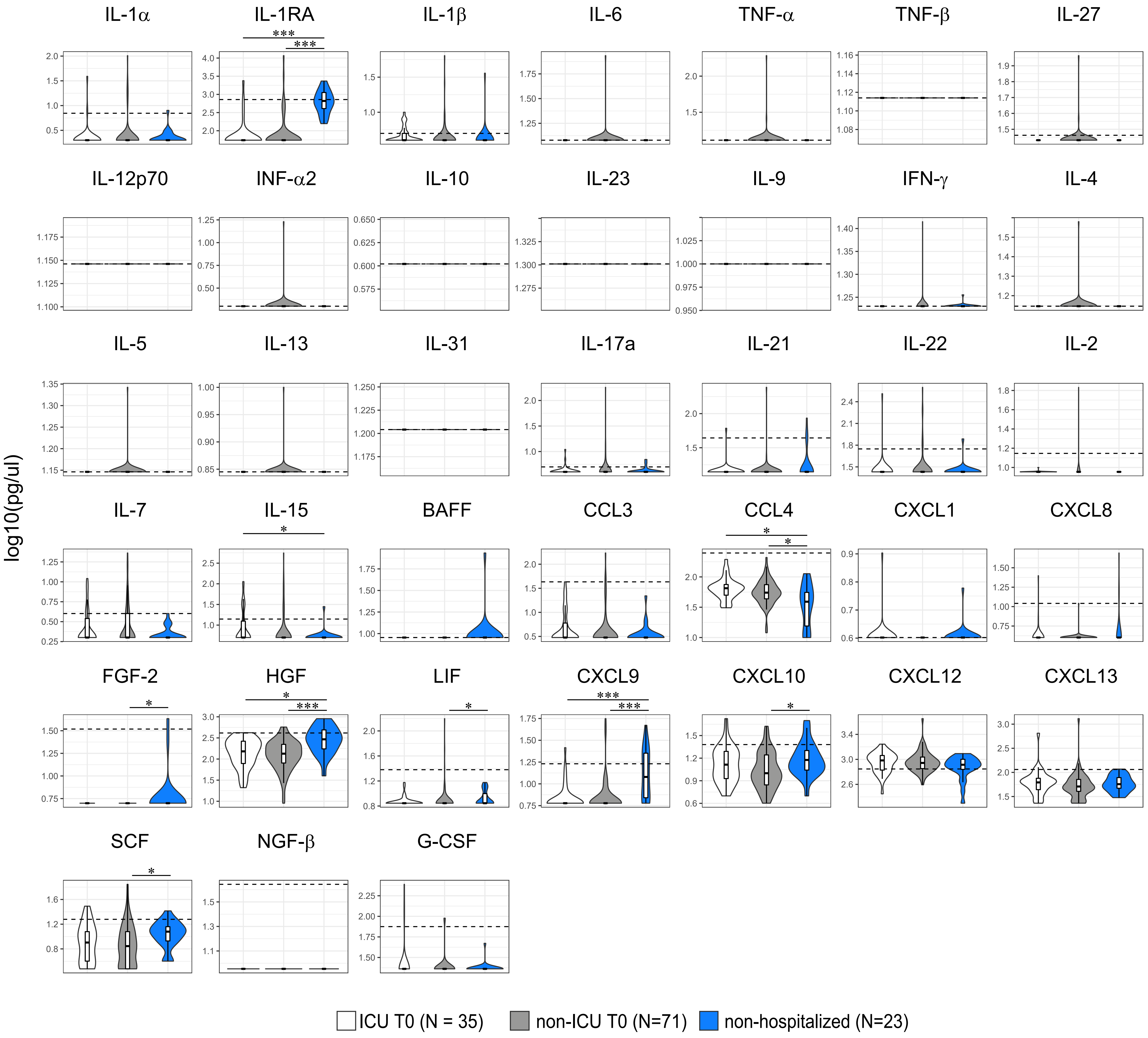

Supplementary Figure S3: Levels of cytokines (IL-1α, IL-1β, IL-6, TNF-α, IL-27, IL-12p70, IFNα2, IL-10, IL-23, IL-9, IFN-γ, IL-4, IL-5, IL-13, IL-31, IL-17A, IL-21, IL-22, IL-2, IL-7, IL-15, BAFF, TNF-β), cytokine receptor (IL-1RA), chemokines (CCL3, CCL4, CXCL1, CXCL8, CXCL9, CXCL10, CXCL12, CXCL13) and growth factors (NGF-β, FGF-2, HGF, LIF, SCF, G-CSF) at 6 months post infection in ICU (N=35), non-ICU (N=71) and non-hospitalized (N=23)). Dotted line represents limit of detection. Statistical significance (*P* values) was obtained using Kruskal-Wallis test, using a Bonferroni correction. *=*P*<0.05; **=*P*<0.01; ***=*P*<0.001.

Supplementary Table S1: Mass cytometry panel

Marker	Isotope	Company	catalogue number
CD45	89	DVS	3089003B
CD45	198	Biolegend	304002
Viability	103Rh	DVS	201103A
IgD	146Nd	DVS	3146005B
CD62L	153Eu	DVS	3153004B
HLA-DR	174Yb	DVS	3174001B
CCR6	141Pr	DVS	3141003A
CD69	144Nd	DVS	3144018B
CD21	152Sm	DVS	3152010B
CD27	155Gd	DVS	3155001B
CD10	158Gd	DVS	3158011B
CD23	164Dy	DVS	3164018B
CD20	171Yb	DVS	3171012B
CD86	156Gd	DVS	3156008B
IgM	172Yb	DVS	3172004B
IgA	148Nd	DVS	3148007B
CD25	149Sm	DVS	3149010B
CD11c	159Tb	DVS	3159001B
CD40	165Ho	DVS	3165005B
CD24	166Er	DVS	3166007B
CD19	142Nd	DVS	3142001B
CD45RA	170Er	DVS	3170010B
CD11b	209Bi	DVS	3209003B
anti PE	145Nd	DVS	3145006B
anti APC	176Yb	DVS	3176007B
anti- FITC	160Gd	DVS	3160011B
CXCR3	154Sm	Biolegend	353718
CD22	161Dy	Biolegend	363502
CD38	163Dy	Biolegend	303535
CD95	168Er	Biolegend	305602
CD3	116Cd	BD	C06795
FcRL4	167Er	Biolegend	340202
IgG	113	BD	555784
CD1c	143Nd	Biolegend	331502
PDL1	147Sm	ThermoFischer	14-5983-82
PDL2	162Dy	ThermoFischer	14-5888-82
CD85J	169Tm	Biolegend	333702
CD34	150Nd	Biolegend	343502
